# Protective effect of complete endoscopic thyroidectomy through different approaches on the recurrent laryngeal nerve

**DOI:** 10.20452/wiitm.2025.17965

**Published:** 2025-07-24

**Authors:** Jianghai Li, Yuming Hua

**Affiliations:** Department of Thyroid and Breast Surgery Affiliated Hospital of Jiangnan University Wuxi, Jiangsu Province China

**Keywords:** endoscopy, protection, recurrent laryngeal nerve, thyroidectomy

## Abstract

**Introduction:**

Papillary thyroid carcinoma (PTC) is a malignancy originating from the thyroid follicular epithelium, accounting for over 80% of thyroid cancer globally. It is often asymptomatic in its early stages, but over time, it may invade the unilateral recurrent laryngeal nerve (RLN), leading to hoarseness, weak speech, or dysphagia. In order to prevent further complications, prompt treatment is required, and surgery remains the primary and most effective option.

**Aim:**

This study aimed to evaluate the protective effect of complete endoscopic thyroidectomy (CET) via different approaches on the RLN.

**Materials and Methods:**

The study analyzed clinical data of 100 patients with clinically node-negative PTC, who underwent CET via the breast approach from February 2023 to February 2025. The patients were divided into 2 groups: the control group (n = 55) which underwent CET via the axillary approach and the study group (n = 45), subjected to CET via the clavicular approach.

**Result:**

Postoperative assessments showed considerable improvements in amplitude perturbation, fundamental frequency perturbation, harmonic-to-noise ratio, and normalized noise energy in both groups, with the study group demonstrating superior results (*P* <⁠0.05). One month after surgery, the voice handicap index score decreased in both groups, as compared with preoperative levels, the study group having obtained a lower score (*P* <⁠0.05). The Scar Cosmesis Assessment and Rating score increased postoperatively in both groups, with a higher score recorded in the study group (*P* <⁠0.05). The Short Form-36 Heath Status Questionnaire scores increased across all dimensions postoperatively in both groups, with the study group showing greater improvements (*P* <⁠0.05).

**Conclusion:**

Compared with the axillary approach, CET via the clavicular approach results in greater postoperative improvements in acoustic parameters, lower voice handicap index scores, better scar appearance, and higher Short Form-36 health status scores.

## INTRODUCTION

Papillary thyroid carcinoma (PTC) accounts for over 80% of thyroid cancer cases and is considered a low-grade malignancy.^1^ PTC is often asymptomatic in its early stages, but as the disease progresses, it may invade the unilateral recurrent laryngeal nerve (RLN), leading to symptoms such as hoarseness, weak speech, or dysphagia. Prompt action is required to prevent further complications,^2^ and surgery remains the primary and most effective treatment for PTC. Traditionally, open thyroidectomy has involved direct excision of the tumor and surrounding tissues through a cervical incision. Although effective, this approach is associated with significant trauma, extended recovery times, high complication rates, and visible scarring, which limits its clinical acceptance.

With advancements in surgical techniques, complete endoscopic thyroidectomy (CET) has gradually replaced conventional open surgery as the preferred treatment for PTC due to its precision, superior cosmetic outcomes, and reduced complication rates.^3^ Various CET approaches exist, with the axillary approach being the most commonly utilized. Nevertheless, this method is associated with extensive subcutaneous dissection, high surgical difficulty, limited exposure of the central region, and potential RLN damage.^4^ In contrast, the clavicular approach is less complex, provides better exposure of the central region, and offers enhanced RLN protection, making it an increasingly popular option.^5^ However, a direct comparison of these 2 approaches is still lacking.

## AIM

This study aimed to compare the effects of CET via the clavicular and axillary approaches on patients with PTC.

## MATERIALS AND METHODS

### Patients

This retrospective study included 100 patients with clinically node-negative PTC who underwent CET via the breast approach at our hospital between February 2023 and February 2025. The patients were divided into 2 groups: the control group (n = 55) which was subjected to CET via the axillary approach) and the study group (n = 45) which underwent CET via the clavicular approach.

### Diagnostic, inclusion, and exclusion criteria

PTC was diagnosed at clinically node-negative stage based on ultrasound-guided fine needle aspiration and histopathological analysis.

The study included patients who voluntarily signed the informed consent form, with stable vital signs, normal immune, coagulation, and cognitive functions, normal emotional state, and good compliance with treatment.

Exclusion criteria involved patients: 1) with neck, chest, or clavicle deformity; 2) with tumor penetrating the posterior capsule or located close to the RLN, or with obvious extraglandular invasion; 3) who could not tolerate surgery due to poor general conditions or severe diseases of other vital systems or organs; 4) with an allergy to anesthetics or intolerance to general anesthesia; 5) with a history of neck surgery or radiotherapy on the affected side; 6) with distant metastases; 7) with tendency to scarring or 8) with undifferentiated carcinoma.

### Anesthesia methods

Intravenous general anesthesia was administered to both groups 30 minutes before surgery. The anesthesia comprised intramuscular injection of atropine sulfate and phenobarbital sodium. The patients were connected to an anesthesia monitor and a noninvasive cardiac function monitor. Anesthesia induction was conducted by intravenous injection of a mixed solution of 0.05 mg/kg midazolam, 2 mg/kg propofol medium and long chain fat emulsion, 0.4 μg/kg sufentanil citrate (Langfang Branch of China National Pharmaceutical Group Industry Co., Ltd., Langfang, China; 10 ml/0.5 mg), and 0.1 mg/kg vecuronium bromide. After successful induction, endotracheal intubation was performed orally for mechanical ventilation. A mixed solution of sufentanil citrate (0.4–0.6 μg/kg/h), propofol medium and long chain fat emulsion (4–12 mg/kg/h), and atrecuonium besylate (0.2–0.4 mg/kg/h) was infused intraoperatively in a target-controlled manner, and the infusion was terminated at skin suture.

### Treatment method for the control group

The control group underwent CET via the axillary approach. The patient was placed in a supine position with the neck reclined and shoulders slightly elevated, while the affected arm was fully extended outward to maximize exposure of the axillary area. The skin, subcutaneous tissue, fascia, muscle, and peritoneum were then incised layer by layer to expose the lateral margin of the pectoralis major muscle.

The pectoralis major muscle was dissected up to the superior clavicular margin along its fascia surface, and the skin flap was elevated using specialized retractors. Dissection continued along the pectoralis major muscle until the sternal and clavicular heads of the sternocleidomastoid muscle were identified. The sternal head was lifted using retractors, the lateral margin of the cervical strap muscles was separated, and the internal jugular vein and common carotid artery were preserved in situ. The scaphoid muscle was dissected, and the anterior cervical strap muscle was retracted upward to fully expose the thyroid lobe on the affected side. A specialized retractor was then secured to the traction bracket to create the surgical cavity.

A 2-cm incision was made at the lateral margin of the breast along the anterior axillary line to serve as the primary operating port, through which a 5-mm endoscope was inserted. A Force-EZ-8c monopolar electrosurgical generator (Valleylab Inc., Minneapolis, Minnesota, United States) was used to dissect, isolate, and seal the middle thyroid vein along the lateral margin of the thyroid gland. The upper pole of the thyroid gland was exposed, and an ultrasonic scalpel was employed to seal the severed thyroid artery and vein at multiple points, while preserving the parathyroid gland and superior laryngeal nerve.

The thyroid capsule was incised using an ultrasonic scalpel, allowing for the separation and protection of the RLN. The inferior pole of the thyroid gland was retracted upward, and the severed inferior thyroid artery and vein were sealed at multiple points using an ultrasonic scalpel. The inferior parathyroid gland was preserved in situ. The tissues and blood vessels between the thyroid gland and trachea were dissected along the lateral trachea using an ultrasonic scalpel, ensuring the RLN remained intact. The thyroid lobe and isthmus were completely excised with an ultrasonic scalpel, maintaining proximity to the contralateral gland.

Finally, hemostasis was performed, the surgical area was cleaned, a drainage tube was placed, and the incision was sutured. The postoperative follow-up was 1 month.

### Treatment method for the study group

The study group underwent CET via the clavicular approach. Following general anesthesia, the patient was positioned supine with the neck reclined and shoulders slightly elevated. The surgeon stood on the affected side, and the assistant, on the opposite side. After disinfection and draping, a primary incision (approximately 3–4 cm in length) was made obliquely along the dermatoglyph, 1 cm below the clavicle on the affected side, with an auxiliary incision (approximately 0.5 cm) made at the edge of the primary incision.

An ultrasonic scalpel was then used to dissect the cervical flap along the deep surface of the platysma, extending upward and toward the midline under endoscopic guidance. A Kirschner wire was inserted through the marginal skin, suspending the cervical flap onto the head frame to create an operative field. A 5-mm endoscope was introduced into this operative field.

Under endoscopic assistance, the sternocleidomastoid muscle was retracted upward, and the lateral margin of the thyroid muscle was dissected to achieve full exposure of the affected thyroid gland. An ultrasonic scalpel was utilized to coagulate and sever the superior thyroid artery and vein at multiple points near the superior pole. Subsequently, the inferior pole and isthmus of the thyroid gland were divided to expose the trachea, followed by the severance of the superior pole vessels.

Dissection of the dorsal and lateral thyroid glands was performed, ensuring the exposure and in-situ protection of the dorsal parathyroid gland and RLN. The thyroid lobe and isthmus were then completely excised, maintaining proximity to the contralateral gland.

Finally, hemostasis was performed, the surgical field was cleaned, a drainage tube was placed, and the incision was sutured. The postoperative follow-up was 1 month.

### Evaluation of indicators

Key intraoperative and postoperative indicators included operation time, intraoperative blood loss, postoperative drainage volume, number of lymph nodes dissected, postoperative length of hospital stay, and the complete exposure rate of the central region. Complete exposure was defined as the ability to fully visualize all boundaries of the central region on endoscopy following dissection.

### Evaluation of parathyroid function

Fasting venous blood samples were collected preoperatively and on postoperative day 7. The serum was separated and analyzed for parathyroid hormone (PTH) and blood calcium levels using ADVIA1200 automated biochemical analyzer and matching reagents (Siemens, Munich, Germany).

### Evaluation of vocal cord function

Vocal cord function was evaluated using a USSA type universal speech spectrum analysis system (Beijing Yangchen Electronic Technology Co., Ltd., Beijing, China) before surgery and 1 month postoperatively. The patients were instructed to sit in a soundproof room (ambient noise <⁠45 dBA), keeping their mouth approximately 15 cm from the microphone in a straight line. Then, they were asked to continuously and stably pronounce the vowel *a*, and the longest phonation time was recorded. A stable 3-second voice segment was selected for analysis from 3 separate recordings. The measurements included amplitude perturbation, fundamental frequency perturbation, harmonic-to-noise ratio, and normalized noise energy.

### Evaluation of voice function

Voice function was assessed using the Voice Handicap Index (VHI) before surgery and 1 month postoperatively.^6^ The VHI includes 10 items across 3 domains: physical, emotional, and functional. Each item was rated on a scale of 0 to 4, with total scores ranging from 0 to 40. Higher scores indicated greater voice impairment.

### Evaluation of cosmetic satisfaction

Cosmetic satisfaction regarding surgical scars was evaluated using the Scar Cosmesis Assessment and Rating score preoperatively and 1 month after the procedure.^7^ The SCAR score ranged from 0 to 15, with lower scores indicating greater satisfaction.

### Assessment of quality of life

Quality of life (QoL)was assessed using the Short Form-36 Health Status Questionnaire (SF-36) before surgery and 1 month postoperatively.^8^ This tool consists of 36 items covering 8 dimensions, each scored from 0 to 100. Higher scores reflect better QoL.

### Observation of complications

Postoperative complications were recorded, including low calcium seizures, postoperative bleeding, incision hematoma, subcutaneous emphysema, incision infection, and lymphorrhagia. These data were used to calculate the overall complication rate.

### Statistical analysis

SPSS 22.0 software (IBM, Armonk, New York, United States) was used for statistical analysis. Measurement data were described as mean (SD), and compared using the independent-samples *t *test between the groups and the paired *t *test within the same group. Count data were expressed as numbers and percentages, and compared using the χ^2 ^test. A *P* value below 0.05 was considered significant.

### Ethics

The study was approved by the ethics committee of the Affiliated Hospital of Jiangnan University (WXJNH2023016).

## RESULTS

### Baseline clinical data

The control group consisted of 30 men and 25 women, aged 26–71 years (mean [SD], 50.84 [3.26] y). Disease duration ranged from 1 to 3 years (mean [SD], 2.1 [0.5] y), and body mass index was 21–25 kg/m² (mean [SD], 22.86 [1.18] kg/m²). Tumor diameters ranged from 1 to 4 cm (mean [SD], 2.2 [0.25] cm). The study group comprised 25 men and 20 women, aged 27–70 years (mean [SD], 50.13 [3.58] y). Disease duration was 1–3 years (mean [SD], 2.12 [0.52] y), and body mass index was 21–25 kg/m² (mean [SD], 22.43 [1.07] kg/m²). Tumor diameters ranged from 1 to 4 cm (mean [SD], 2.22 [0.23] cm). Baseline characteristics of the 2 groups were comparable (*P* >0.05).

### Surgery-related indicators

No differences were observed between the study and control groups regarding postoperative drainage volume and the number of lymph nodes dissected (*P* >0.05). However, the study group had shorter operation time, reduced intraoperative blood loss, and a higher rate of complete exposure of the central region than the control group (*P* <⁠0.05; TABLE 1).

**TABLE 1:** Surgery‑related indicators

Parameter	Control group (n = 55)	Study group (n = 45)	χ2**/***t*	*P* value
Operation time, min	110.86 (10.25)	89.49 (8.99)	10.955	<⁠0.001
Intraoperative blood loss, ml	64.35 (6.84)	43.84 (5.52)	16.243	<⁠0.001
Postoperative length of stay, d	6.53 (1.35)	4.25 (0.96)	9.525	<⁠0.001
Complete exposure rate of central region, n (%)	46 (83.64)	44 (97.78)	4.04	0.04
Postoperative drainage volume, ml	95.25 (10.25)	94.24 (11.26)	0.469	0.64
Number of lymph nodes dissected, n (%)	2.93 (0.82)	2.9 (0.8)	0.184	0.85

Data are presented as mean (SD) unless indicated otherwise.

### Parathyroid function

Preoperatively, there were no differences in serum PTH and blood calcium levels between the groups (*P* >0.05). At 7 days postoperatively, both groups saw a decrease in these levels, as compared with the preoperative values. PTH value in the control group was 3.71 ng/l (*P *<⁠0.001), and in the study group, it was 8.25 ng/l (*P *<⁠0.001). Calcium level in the study group was 7.62 mmol/l, whereas in the control group, it was 20.16 mmol/l (*P *<⁠0.001). However, the study group maintained higher levels of calcium than the control group (*P* <⁠0.05; TABLE 2).

**TABLE 2:** Parathyroid function

Parameter	Time point	Control group (n = 55)	Study group (n = 45)	*t* value	*P* value
PTH, ng/l	Preoperatively	41.02	40.32	0.714	0.48
		(5.32)	(4.28)		
	7 Days postoperatively	20.32 (5.52)a	33.65 (4.65)a	12.883	<0.001
Blood	Preoperatively	2.36	2.39	0.447	0.66
calcium, mmol/l		(0.32)	(0.35)		
	7 Days postoperatively	1.32 (0.25)a	2.05 (0.28)a	13.762	<0.001

Data are presented as mean (SD).

**a ***P* <⁠0.05 vs the same group preoperatively

Abbreviations: PTH, parathyroid hormone

### Vocal cord function

Preoperatively, there were no intergroup differences in amplitude perturbation, fundamental frequency perturbation, harmonic-to-noise ratio, or normalized noise energy (*P* >0.05). Postoperatively, both groups exhibited improvements in these parameters, as compared with their preoperative values, with the study group showing better outcomes than the control group (*P* <⁠0.05; TABLE 3).

**TABLE 3:** Vocal cord function

Parameter	Time point	Control group (n = 55)	Study group (n = 45)	*t* value	*P* value
Amplitude	Preoperatively	4.77	4.72	0.586	0.56
perturbation, %		(0.42)	(0.43)		
	1 Month postoperatively	3.05 (0.35)a	2.37 (0.15)a	12.144	<0.001
Fundamental	Preoperatively	0.76	0.78	0.387	0.7
frequency		(0.27)	(0.24)		
perturbation, %	1 Month postoperatively	0.46 (0.15)a	0.23 (0.14)a	7.859	<0.001
Harmonic‑to‑	Preoperatively	15.63	15.64	0.012	0.99
‑noise ratio, dB		(4.21)	(4.13)		
	1 Month postoperatively	20.42 (5.13)a	25.21 (5.09)a	4.662	<0.001
Normalized noise energy,	Preoperatively	–5.02 (0.72)	–5.05 (0.74)	0.205	0.84
dB 1 Month postoperatively	–13.02 (2.58)a	–20.84 (3.77)a	12.272	<0.001	

Data are presented as mean (SD).

**a ***P* <⁠0.05 vs the same group preoperatively

### Voice function

Preoperatively, there were no differences in the VHI scores between the groups (*P* >0.05). At 1 month postoperatively, both groups showed a decrease in VHI scores, as compared with the preoperative values, with the study group exhibiting significantly lower scores than the control group (*P* <⁠0.05; TABLE 4).

**TABLE 4:** Voice function, assessed with the Voice Handicap Index

Time point	Control group (n = 55)	Study group (n = 45)	*t* value	*P* value
Preoperatively	25.86 (5.25)	24.89 (5.29)	0.916	0.36
1 Month postoperatively	18.35 (4.84)	10.84 (3.52)	8.693	<0.001
*t* value	7.8	14.833	–	–
*P* value	<0.001	<0.001	–	–

Data are presented as mean (SD).

### Cosmetic satisfaction

Preoperatively, there were no intergroup differences in the SCAR scores (*P* >0.05). At 1 month postoperatively, both groups exhibited higher SCAR scores than before surgery, with the study group scoring better than the control group (*P* <⁠0.05; TABLE 5).

**TABLE 5:** Cosmetic satisfaction, as per the Scar Cosmesis Assessment and Rating score

Time point	Control group (n = 55)	Study group (n = 45)	*t* value	*P* value
Preoperatively	6.8 (1.39)	6.79 (1.41)	0.036	0.97
1 Month postoperatively	9.35 (0.84)	11.34 (0.52)	13.86	<0.001
*t* value	11.644	20.31	–	–
*P* value	<0.001	<0.001	–	–

Data are presented as mean (SD).

### Quality of life

Preoperatively, there were no differences in the SF-36 scores across all dimensions between the study and control groups (*P* >0.05). At 1 month postoperatively, both groups exhibited higher SF-36 scores in all dimensions, as compared with the preoperative values, with the study group outscoring the control group (*P* <⁠0.05; TABLE 6).

**TABLE 6:** Quality of life assessed with the Short Form‑36 Health Status Questionnaire

Domain		Control group (n = 55)	Study group (n = 45)	t value	P value
Physical functioning	Preoperatively	44.55 (4.81)	43.75 (5.71)	0.761	0.45
	1 Month postoperatively	72.56 (8.35)ᵃ	80.65 (9.34)ᵃ	4.569	<0.001
Bodily pain	Preoperatively	68.55 (4.25)	68.44 (4.25)	0.129	0.9
	1 Month postoperatively	72.44 (8.72)ᵃ	81.32 (9.43)ᵃ	4.884	<0.001
Role physical	Preoperatively	53.45 (5.62)	52.43 (5.85)	0.887	0.38
	1 Month postoperatively	75.46 (8.85)ᵃ	84.64 (9.25)ᵃ	5.057	<0.001
Role emotional	Preoperatively	52.44 (6.35)	51.96 (5.85)	0.39	0.7
	1 Month postoperatively	71.25 (8.04)ᵃ	80.63 (9.02)ᵃ	5.494	<0.001
Social functioning	Preoperatively	39.02 (4.43)	38.73 (4.34)	0.329	0.74
	1 Month postoperatively	65.48 (7.45)ᵃ	73.76 (8.54)ᵃ	5.176	<0.001
Mental health	Preoperatively	63.25 (7.06)	63.52 (7.34)	0.187	0.85
	1 Month postoperatively	72.24 (8.35)ᵃ	81.25 (7.24)ᵃ	5.695	<0.001
Vitality	Preoperatively	32.54 (3.62)	32.25 (3.83)	0.388	0.7
	1 Month postoperatively	73.31 (8.42)ᵃ	81.02 (9.14)ᵃ	4.383	<0.001
General health	Preoperatively	36.28 (4.34)	35.52 (4.11)	0.892	0.38
	1 Month postoperatively	70.24 (8.43)ᵃ	78.38 (9.05)ᵃ	4.647	<0.001

Data are presented as mean (SD).

**a ***P* <⁠0.05 vs the same group preoperatively

### Incidence rate of complications

The incidence rate of complications in the study group was not different from that registered in the control group (*P* >0.05; TABLE 7).

**TABLE 7:** Incidence rate of complications

Complication	Control group (n = 55)	Study group (n = 45)	χ2	*P* value
Hypocalcemic seizures	1 (1.82)		–	–
Postoperative bleeding	1 (1.82)	1 (2.22)	–	–
Incision hematoma	1 (1.82)	1 (2.22)	–	–
Incision infection			–	–
Lymphorrhagia			–	–
Subcutaneous emphysema			–	–
Total	3 (5.45)	2 (4.44)	0.053	0.82

Data are presented as numbers (percentages).

## DISCUSSION

PTC is not only one of the fastest-growing solid malignancies in recent years, but also the most common malignancy in the head and neck region. Surgery is the treatment of choice for PTC.^9^ However, due to the complex anatomy of the thyroid gland, there is a high risk of inadvertently damaging the parathyroid gland and RLN during surgery. This can lead to a decline in parathyroid function and RLN injury postoperatively, which severely impacts rehabilitation and QoL. Therefore, protecting the parathyroid gland and RLN during surgery is critical. Traditional radical thyroidectomy has long been used for treating PTC. Although it effectively removes the thyroid cancer focus, it is associated with significant intraoperative trauma and a high incidence of postoperative complications.^10^ In contrast, CET is a minimally-invasive technique currently favored for PTC, and its outcomes are closely linked to the surgical approach, that is, the clavicular vs axillary approach.

In our study, postoperative drainage volume and the number of lymph nodes dissected were similar between the study and control groups. However, the study group demonstrated a shorter operation time, reduced intraoperative blood loss, and a higher rate of complete central region exposure in comparison with the control group. These findings indicate that CET via the clavicular approach can effectively reduce operation time and postoperative hospital stay, minimize blood loss, and improve exposure of the central region, relative to the axillary approach. This advantage may be due to the fact that the axillary approach requires cutting through the anterior cervical muscle group, thereby increasing surgical trauma. In contrast, the clavicular approach offers a shorter operating distance and is less influenced by the sternoclavicular joint, which reduces surgical difficulty and trauma while better exposing the central region.^11^;^12^ Moreover, although both approaches allow for thorough dissection of the central region, the clavicular approach achieves the same effect in a shorter time, thus shortening the overall operation time.^13^;^14^

At 1 month postoperatively, the SCAR score increased in both groups, as compared with preoperative values. However, the study group scored higher than the control group. This suggests that CET via both approaches decreases cosmetic satisfaction, particularly with the clavicular approach. This outcome may be attributed to the fact that subclavian muscle tissue is more compact than subaxillary tissue, making incisions in this area more prone to prominent scarring due to increased tension.^15^

Hypoparathyroidism and RLN injury are common complications following CET, potentially compromising surgical outcomes and hindering postoperative rehabilitation. The parathyroid gland, an essential endocrine organ, primarily secretes PTH, which regulates calcium metabolism, maintains blood calcium homeostasis, and supports normal metabolic functions. The RLN controls vocal cord movement, and injury to this nerve can result in the loss of innervation of the internal laryngeal muscles on the affected side, leading to swallowing dysfunction, dysphonia, vocal cord dysfunction, dyspnea, and even asphyxia.^16^;^17^

In this study, postoperative measures, including amplitude perturbation, fundamental frequency perturbation, harmonic-to-noise ratio, and normalized noise energy, were all improved in comparison with preoperative values in both groups, with the study group outperforming the control group. Similarly, both groups exhibited a reduction in VHI scores at 1 month postoperatively, with the study group obtaining a considerably lower score than the control group. These findings suggest that CET via the clavicular approach offers better protection of the parathyroid gland and RLN than the axillary approach. This benefit may be attributed to the clearer surgical field provided by the clavicular approach, which allows for improved visualization of the thyroid and parathyroid glands and their supplying vessels, thereby facilitating their preservation.^18^ Additionally, since the clavicular approach is less impacted by the sternoclavicular joint, it offers a better-exposed surgical field. This enables more precise differentiation and identification of nerves and vessels, thereby reducing the likelihood of RLN injury.^19^;^20^

Furthermore, at 1 month postoperatively, the SF-36 scores across all dimensions increased in both groups in comparison with preoperative values, with the study group achieving markedly higher scores than the control group. This suggests that the clavicular approach not only better preserves the RLN and parathyroid function but also enhances overall QoL. Finally, the incidence of complications was comparable between the 2 approaches, with rates of 4.44% (2/45) in the study group and 5.45% (3/55) in the control group, demonstrating that both CET approaches have a satisfactory safety profile.

Nevertheless, this study has certain limitations. As this was a retrospective study, randomization was not applicable. This real-world allocation method is consistent with another retrospective study evaluating different endoscopic thyroidectomy approaches.^21^ This may introduce selection bias, so prospective randomized studies are needed to further validate our findings.

### CONCLUSIONS

In conclusion, compared with CET via the axillary approach, CET via the clavicular approach is characterized by a shorter operation time, reduced postoperative hospital stay, lower intraoperative blood loss, and better exposure of the central region. This approach effectively protects parathyroid function and the RLN, resulting in a higher postoperative QoL.
